# α-Aminobutyric Acid Constrains Macrophage-Associated Inflammatory Diseases through Metabolic Reprogramming and Epigenetic Modification

**DOI:** 10.3390/ijms241310444

**Published:** 2023-06-21

**Authors:** Fei Li, Yuting Xia, Shijie Yuan, Xiaorong Xie, Lin Li, Yuan Luo, Qiuyang Du, Yuqi Yuan, Ran He

**Affiliations:** 1 Department of Immunology, School of Basic Medicine, Tongji Medical College, Huazhong University of Science and Technology, Wuhan 430032, China; feili@hust.edu.cn (F.L.);; 2 Department of Dermatology, Tongji Medical College, Huazhong University of Science and Technology, Wuhan 430032, China; 3 State Key Laboratory of Oncology in South China, Sun Yat-sen University Cancer Center, Guangzhou 510060, China

**Keywords:** macrophage, inflammation, α-aminobutyric acid, metabolic reprogramming, EZH2, H3K27me3

## Abstract

Metabolites play critical roles in macrophage polarization and in their function in response to infection and inflammation. α-aminobutyric acid (AABA), a non-proteinogenic amino acid which can be generated from methionine, threonine, serine, and glycine, has not been studied extensively in relation to macrophage polarization and function. In this study, we aimed to investigate the immunomodulatory function of AABA in regulating M1 macrophage polarization and function in vitro and in vivo. We stimulated bone-marrow-derived macrophages with lipopolysaccharide (LPS) to generate M1 macrophages. Subsequently, we induced sepsis and colitis in mice, followed by treatment with AABA. We then analyzed the samples using ELISA, real-time PCR, Western blotting, flow cytometry, and histopathological analysis to evaluate cytokine secretion, inflammatory gene expression, macrophage activation, disease progression, and inflammation severity. Additionally, metabolomic and chromatin immunoprecipitation-qPCR were conducted to investigate the function of AABA on metabolic reprogramming and epigenetic modifications of M1 macrophages. Our results revealed that AABA inhibited M1 macrophage polarization and function, which led to prolonged survival in septic mice and reduced disease severity in colitis mice. Mechanically, AABA promoted oxidative phosphorylation (OXPHOS) and glutamine and arginine metabolism while inhibiting glycolysis. Moreover, AABA could increase the occupancy of trimethylation of histone H3K27 at the promoter regions of M1 macrophage-associated inflammatory genes, which contributed to the inhibition of M1 macrophage polarization. These findings suggest that AABA may have therapeutic potential for inflammatory diseases by regulating macrophage polarization and function through metabolic and epigenetic pathways.

## 1. Introduction

Macrophages are the forefront cells in innate immunity and assume a pivotal part in the early promotion and resolution of inflammation [1]. The polarization and activation of macrophages are highly plastic and dynamic, and can be manipulated by different microenvironmental signals, thus exercising distinct functions that are relevant to a variety of diseases [2,3]. Activated by LPS or other pathological cues, M1 macrophages are portrayed by the massive emission of pro-inflammatory cytokines (e.g., tumor necrosis factor alpha [TNF-α] and interleukin [IL]-6), abundant production of reactive nitrogen and oxygen intermediates (e.g., nitric oxide [NO] and responsive oxygen intermediates) [4]. M2 macrophages stimulated by IL-4/IL-13, on the other hand, are considered crucial for parasite containment, tissue healing, and tumor growth [2,5]. However, the over-activation or sustained activation of macrophages in response to inflammatory diseases, such as sepsis and inflammatory bowel disease, can cause tissue damage and worsen the disease [6,7]. Such properties make macrophages important during the progression of M1 macrophage-associated inflammatory diseases. Therefore, macrophages are an important target for treating inflammation-related diseases [8].

Macrophage polarization involves the coordination of transcription factor networks, epigenetic modifications, and cellular metabolism [3,9,10]. To accommodate changes in cellular function, intracellular metabolic patterns shift dramatically during macrophage polarization. Glycolysis is a hallmark of pro-inflammatory macrophages (M1), whereas anti-inflammatory macrophages (M2) mainly depend on OXPHOS [11]. There is increasing evidence that metabolic reprogramming triggered by metabolites modulates macrophage polarization and function [12]. NO constrains OXPHOS and impedes plasticity in M1 macrophages [13]. The accumulation of itaconic acid inhibits the succinate dehydrogenase (SDH), which blocks the production of reactive oxygen species (ROS), further inhibiting the hypoxia-inducible factor (HIF)-1a activity and interleukin (IL)-1b production [14]. Moreover, there is increasing evidence supporting the view that amino acid metabolism can direct macrophage polarization and function [15,16,17]. Glycine metabolism inhibits M1 macrophage polarization [18], whereas serine metabolism facilitates IL-1β secretion in M1 macrophages [19]. Furthermore, exogenous methionine and serine are involved in LPS-stimulated macrophage-associated inflammation by enhancing S-adenosylmethionine (SAM) production [20]. LPS is known to stimulate M1 macrophage activation via the canonical signaling pathway of mitogen-activated protein kinase (MAPK) and nuclear factor (NF)-κB [21]. However, macrophage polarization is synergistically regulated by alternative mechanisms, including epigenetic and metabolic reprogramming, in addition to signaling cascades. For example, lactate stimulates the expression of the M2 macrophage marker gene *Arginase1* by increasing histone lactylation [22], and alpha-ketoglutaric acid (α-KG) represses the trimethylation of histone H3 K27 (H3K27me3) at the promoters of M2 marker genes to promote M2 polarization [23]. Additionally, one-carbon metabolism modulates the M1 macrophages’ inflammatory response by affecting the availability of SAM, a primary methyl donor for histone methylation [20]. Thus, metabolites can target histone modification to modulate macrophage polarization. 

AABA is a non-proteinogenic amino acid generated as a byproduct of either cysteine biosynthesis or the metabolic pathways of methionine, threonine, serine, and glycine [24,25]. Previous studies have reported that AABA supports cardiomyocyte activity by increasing glutathione (GSH) production [25], and plasma AABA level is associated with depressive symptoms and the progression of sepsis [24,26]. However, the relationship between AABA and M1 macrophage polarization and function has never been investigated. 

In the present study, we determined the level of AABA in LPS-stimulated macrophages. We had two research objectives. The first was to investigate the effects of AABA on M1 macrophage activation and polarization to determine its role in macrophage metabolic reprogramming. The second was to explore the function of AABA in M1 macrophage-associated inflammatory disease using an LPS- and cecum-ligation-and-puncture (CLP)-induced sepsis model and a dextran-sulfate-sodium (DSS)-induced colitis model. Our findings provide important evidence for the potential usefulness of AABA as an effective intervention against inflammatory diseases.

## 2. Results

### 2.1. LPS Stimulation Impacts AABA Metabolism in Macrophages

**Figure 1:**
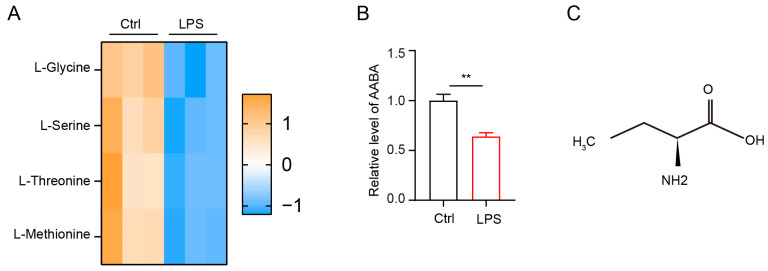
LPS induces a notable decrease in AABA in macrophages. (**A**) The relative level of methionine, threonine, serine, and glycine. (**B**) The relative level of AABA. (**C**) The chemical formula of AABA. ** *p* < 0.01.

Amino acid metabolism profoundly affects macrophage polarization [15]. To investigate the metabolic changes in amino acids following LPS stimulation, we collected bone-marrow-derived macrophages (BMDMs) stimulated with 100 ng/mL LPS for 36 h and then evaluated amino acid level using liquid chromatography-tandem mass spectrometry (LC-MS/MS). Our data showed that the level of methionine, threonine, serine, and glycine markedly decreased after LPS stimulation (Figure 1A). As previously reported, AABA is a common byproduct of methionine, threonine, serine, and glycine [24]. Therefore, we analyzed the level of AABA in LPS-stimulated macrophages and found that it decreased significantly after LPS stimulation (Figure 1B). AABA is a nonpolar amino acid containing four carbons and is composed of an amino group (NH2), a methyl group (CH3), an ethylene group (CH2), and a carboxyl group (COOH) (Figure 1C). These data indicate that LPS stimulation inhibits AABA biosynthesis in macrophages.

### 2.2. AABA Impairs Activation and Function of M1 Macrophages

**Figure 2:**
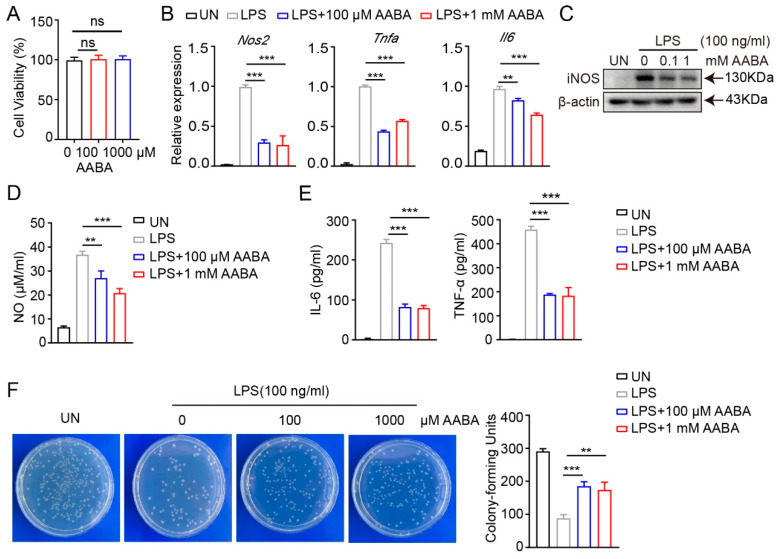
AABA inhibited LPS-induced macrophage activation. (**A**) Cell viability. (**B**) The mRNA levels of *Nos2*, *Tnfa*, and *Il6*. (**C**) Western blot image of iNOS. (**D**) NO level. (**E**) IL-6 and TNF-α levels. (**F**) The representative plots of colony (left) and colony counts (right). ** *p* < 0.01, *** *p* < 0.001, ns: no significance.

From the above results, we found that the polarization of M1 macrophages induced by LPS stimulation caused a decrease in AABA level, suggesting a close association between AABA and M1 macrophage polarization. Since macrophages assume a crucial part in the pathogenesis of sepsis by releasing abundant pro-inflammatory mediators and cytokines [1,2], we speculated that AABA may affect the inflammatory function of macrophages. Therefore, we activated BMDMs with 100 ng/mL LPS in vitro and added 100 μM or 1 mM AABA to the medium, then evaluated cell viability. Although AABA treatment had no effects on cell viability (Figure 2A), the expression of the inflammation-associated genes *Nos2*, *Tnfa*, and *Il6* was obviously suppressed with AABA treatment (Figure 2B). In line with these data, AABA also suppressed the protein expression of inducible nitric oxide synthase (iNOS) in LPS-activated macrophages (Figure 2C). The enzyme-linked immunosorbent assay (ELISA) analysis of LPS-stimulated macrophages demonstrated that AABA treatment effectively reduced the LPS-induced production of NO (Figure 2D). Furthermore, M1 macrophages released high levels of IL-6 and TNF-α after stimulation with LPS, while the administration of AABA dramatically decreased NO production (Figure 2E). 

To evaluate the bactericidal capacity of AABA-treated macrophages, we stimulated with LPS alone or LPS plus AABA for 24 h, followed by co-culture with *Escherichia coli* for 6 h. AABA treatment inhibited the bactericidal activity of the M1 macrophages (Figure 2F). Collectively, these results prompt that AABA can repress the polarization and function of M1 macrophages in vitro. 

### 2.3. AABA Protects Mice from LPS-Induced Sepsis

**Figure 3:**
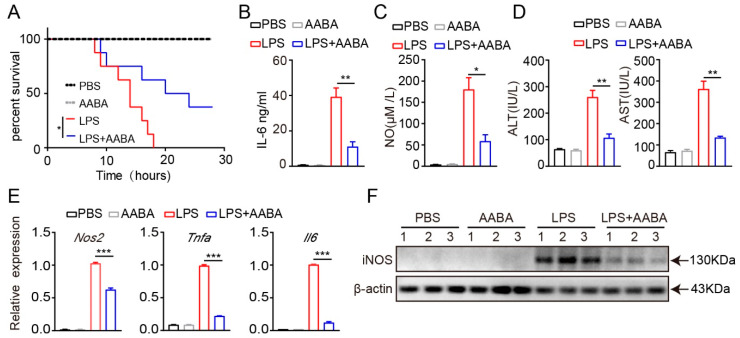
AABA relieves LPS-induced inflammation response. (**A**) Survival rate. (**B**) Sera IL-6. (**C**) Sera NO. (**D**) Sera transaminase ALT and AST. (**E**) The *Nos2*, *Tnfa*, and *Il6* mRNA levels of peritoneal macrophages. (**F**) Western blot image of iNOS of peritoneal macrophages. * *p* < 0.05, ** *p* < 0.01, *** *p* < 0.001.

LPS produced by Gram-negative bacteria induces sepsis by initiating an overactive inflammatory response [27]. Injection of LPS can quickly activate macrophages, which release pro-inflammatory cytokines such as TNF-α and IL-6, that are crucial for the occurrence and maintenance of the body’s overall inflammatory process [28]. In addition, LPS also stimulates macrophages to produce a series of chemical substances, such as NO and various proteases, which also play an important regulatory role in the process of inflammation [29]. To determine the function of AABA in the systemic pro-inflammatory response, we treated LPS-induced septic mice with AABA. Mice were intraperitoneally injected with 20 mg/kg LPS, followed by an intraperitoneal injection of 75 mg/kg AABA. The results showed that AABA administration effectively prolonged the survival of LPS-treated mice (Figure 3A). To further discover the effects of AABA on the secretion of inflammatory cytokines and mediators induced by LPS, sera were collected 6 h after LPS administration, with or without AABA treatment, and subjected to ELISA. AABA treatment attenuated the LPS-induced production of IL-6, concomitant with a decreased NO level (Figure 3B,C). AABA treatment also ameliorated LPS-induced liver damage as measured by an aspartate aminotransferase (AST) and alanine aminotransferase (ALT) ELISA kit (Figure 3D). In addition, the *Nos2*, *Tnfa*, and *Il6* mRNA expression and iNOS protein level of peritoneal macrophages from mice treated with AABA were notably downregulated (Figure 3E,F). These results together demonstrate that AABA protects mice from LPS-induced sepsis by constraining the M1 macrophage inflammatory response.

### 2.4. AABA Relieves the Development of CLP-Induced Sepsis

**Figure 4:**
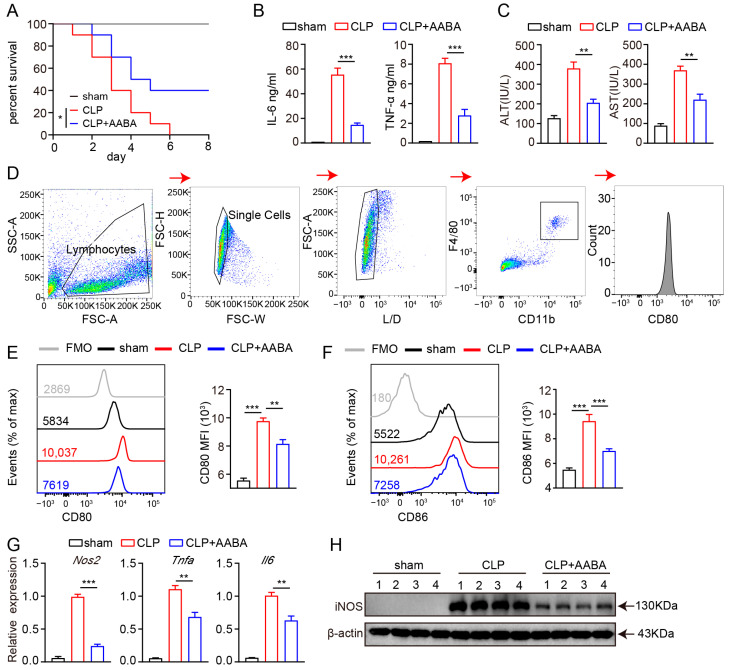
AABA relieves CLP-induced inflammation response. (**A**) Survival rate. (**B**) Sera TNF-α and IL-6. (**C**) Sera transaminase AST and ALT. (**D**) Flow cytometry gating strategy for peritoneal macrophages. (**E**) Representative flow plots (left) and MFI analysis (right) of CD80. (**F**) Representative flow plots (left) and MFI analysis (right) of CD86. (**G**) The *Nos2*, *Tnfa*, and *Il6* mRNA levels of peritoneal macrophages. (**H**) Western blot image of iNOS. FMO: fluorescence minus one, MFI: mean fluorescence intensity. * *p* < 0.05, ** *p* < 0.01, *** *p* < 0.001.

We further explored the potential therapeutic role of AABA in treating inflammatory diseases using a CLP-induced sepsis model, given that this model has been found to bear higher similarity than the LPS-induced sepsis model to human sepsis in terms of immune, inflammatory, and biochemical alterations [30]. The mice were divided into three groups: sham, CLP + PBS, and CLP + AABA. After the CLP procedure, the CLP + AABA group was treated with 75 mg/kg AABA. We saw that AABA administration effectively prolonged the survival time of CLP-treated mice (Figure 4A) and attenuated the secretion of IL-6 and TNF-α induced by the CLP procedure (Figure 4B). Consistently, AABA treatment effectively alleviated liver damage, as indicated by decreased AST and ALT levels in mice sera (Figure 4C). Flow cytometric analysis of CD80 and CD86 in peritoneal macrophages further revealed that AABA treatment markedly inhibited their activation following the CLP procedure (Figure 4D–F). Consistent with these observations, AABA treatment led to a reduction in *Nos2*, *Tnfa*, and *Il6* mRNA levels, as well as a decrease in the iNOS protein expression of peritoneal macrophages (Figure 4G,H). Collectively, our data suggest that AABA can protect against CLP-induced septic shock and suppress inflammatory responses in mice.

### 2.5. AABA Protects Mice against DSS-Induced Inflammatory Bowel Disease

**Figure 5:**
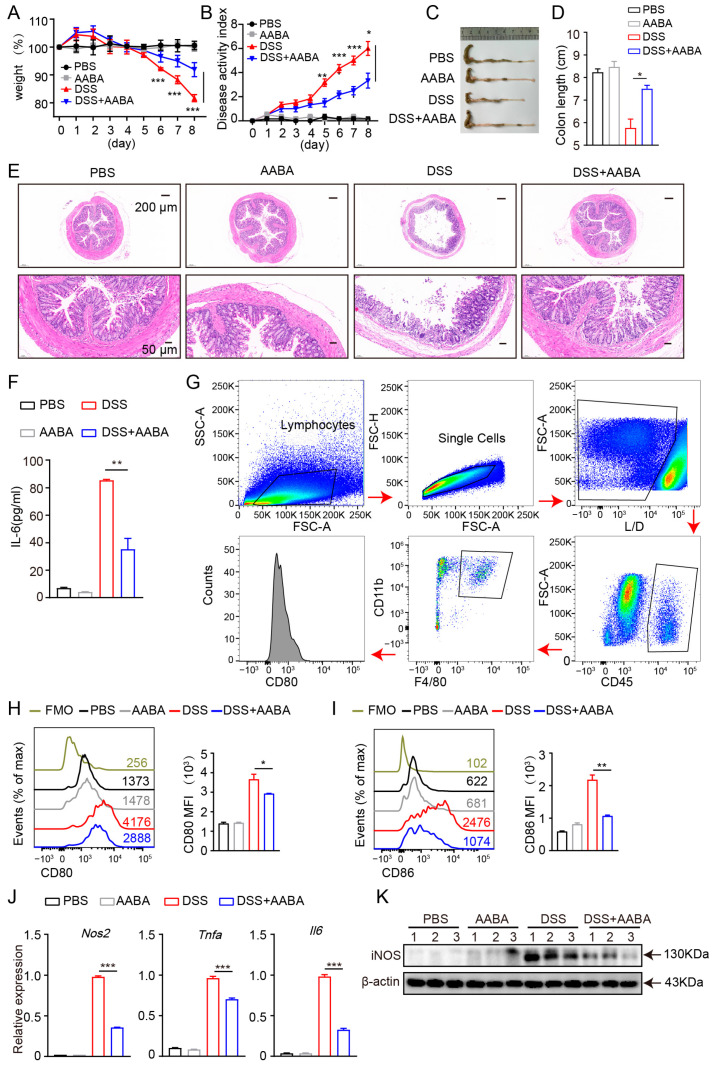
AABA protects against a DSS-induced inflammatory bowel disease in mice. (**A**) Percentage of body weight. (**B**) Disease activity index. (**C**) The representative images of colon. (**D**) Colon length. (**E**) The representative H&E-stained images of colon sections. (**F**) Sera IL-6. (**G**) Flow cytometry gating strategy for intestinal cells. (**H**) Representative flow plots (left) and MFI analysis (right) of CD80. (**I**) Representative flow plots (left) and MFI analysis (right) of CD86. (**J**) The *Nos2*, *Tnfa*, and *Il6* mRNA levels of F4-80^+^ CD11b^+^ macrophages. (**K**) Western blot image of iNOS of F4-80^+^ CD11b^+^ macrophages. FMO: fluorescence minus one, MFI: mean fluorescence intensity. * *p* < 0.05, ** *p* < 0.01, *** *p* < 0.001.

Macrophages are thought to drive DSS-induced colitis by releasing pro-inflammatory chemokines and cytokines [7]. Therefore, we further evaluated the protective effects of AABA on the development of DSS-induced colitis in mice. Starting from the day of 2.5% DSS administration, the mice were injected intraperitoneally with 75 mg/kg AABA for 8 days. Administration of AABA inhibited body weight loss induced by DSS, whereas no significant impact was observed on the body weight of healthy mice (Figure 5A). Furthermore, AABA-treated mice displayed considerably lower disease activity index (DAI) scores and resisted colonic shortening on day 8 following DSS exposure compared with control mice (Figure 5B–D). Consistent with the alleviated clinical signs, AABA-treated mice showed milder histological damage, as evidenced by less disruption of epithelial layers, better preservation of intestinal crypts, and less submucosal inflammatory cell infiltration relative to control DSS-exposed mice (Figure 5E). 

Next, we examined the secretion of IL-6 in mice sera. The outcomes demonstrated that AABA treatment notably attenuated DSS-induced IL-6 secretion (Figure 5F). Moreover, flow cytometric analysis showed that the expression of the co-stimulatory molecules CD80 and CD86 of intestinal macrophages was inhibited by AABA (Figure 5G–I). Furthermore, intestinal macrophages from AABA-treated mice showed a prominent downregulation in *Nos2*, *Tnfa*, and *Il6* mRNA expression and iNOS protein level (Figure 5J,K). These data suggest that AABA effectively relieves DSS-induced inflammatory bowel disease by inhibiting the activation and function of pro-inflammatory macrophages.

### 2.6. AABA Reprograms Metabolism of M1 Macrophages

**Figure 6:**
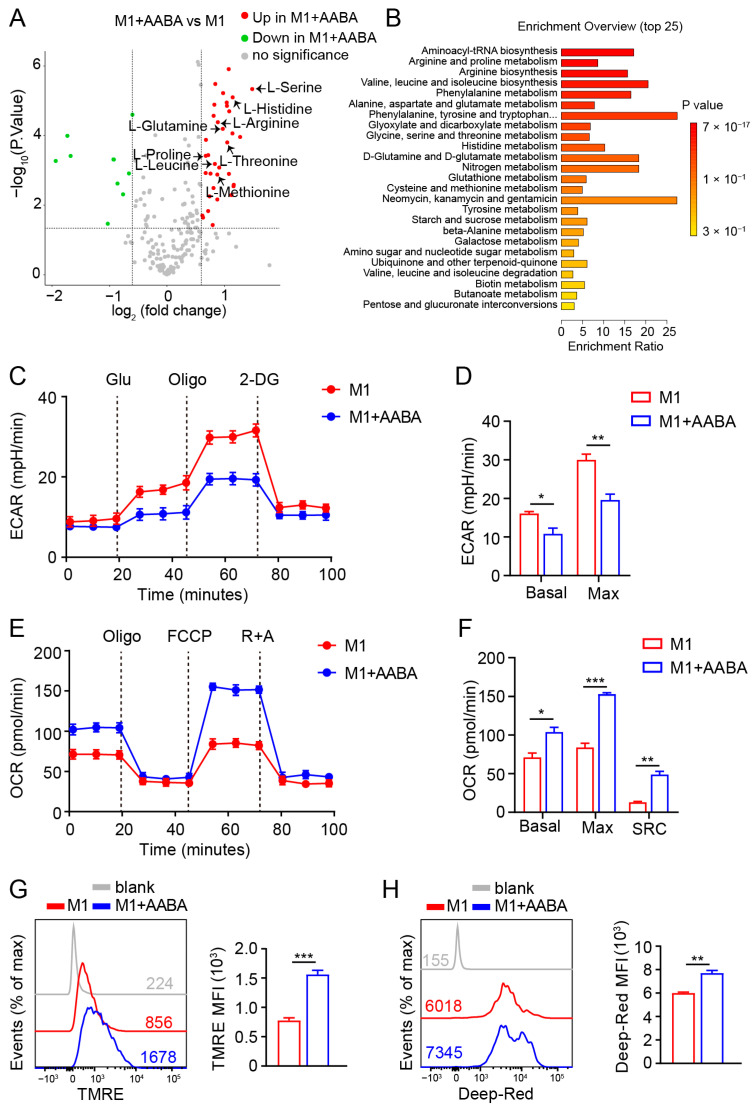
AABA reprograms M1 macrophage metabolism. (**A**) Metabolite LC-MS/MS analysis. fold change > 1.5, *p* < 0.05. (**B**) KEGG analysis. (**C**) ECAR analysis. (**D**) The basal and max ECAR. (**E**) OCR analysis. (**F**) The basal, max OCR, and spare respiration capacity (SRC). (**G**) Representative flow plots of TMRE (left) and the MFI analysis (right). (**H**) Representative flow plots of Mito Tracker Deep Red (left) and the MFI analysis (right). MFI: mean fluorescence intensity. * *p* < 0.05, ** *p* < 0.01, *** *p* < 0.001.

The immune metabolic state of macrophages can affect their polarization status, ultimately impacting their related functions [10]. Investigating the crosstalk between immunometabolism and polarization in macrophages can facilitate a deeper comprehension of their model of action, as well as offering new insights for treating inflammatory diseases. In order to assess how immunometabolism affects the polarization status of macrophages and its potential significance in managing inflammatory diseases, we investigated the metabolic effects of administering AABA on M1 macrophages. Our analysis revealed that AABA treatment increased levels of several key amino acids, including arginine, serine, methionine, threonine, and glutamine (Figure 6A). These changes were further supported by KEGG analysis, which showed that AABA enhanced the arginine, glutamine, and glutamate metabolism pathways (Figure 6B). As a well-known trigger of macrophage glycolysis, LPS stimulation is known to promote inflammatory function by increasing the production of crucial factors [31,32]. However, our data showed that AABA treatment had the opposite effect of decreasing the basal and max extracellular acidification rate (ECAR) triggered by LPS (Figure 6C,D). This contrast was further supported by data indicating that AABA treatment notably increased the basal and max oxygen consumption rate (OCR) in LPS-activated macrophages, consistent with an increase in the spare respiratory capacity (SRC) of the cells (Figure 6E,F). Notably, AABA treatment also resulted in increased mitochondrial membrane potential, as indicated by the enhanced fluorescence of potentially sensitive tetramethylrhodamine ethyl ester (TMRE) and Mito Tracker Deep Red (Figure 6G,H), suggesting that AABA profoundly regulates critical metabolic pathways in LPS-stimulated macrophages. 

### 2.7. AABA Enhances EZH2 Expression to Improve H3K27me3 Chromatin Occupancy in M1 Macrophage-Associated Inflammatory Genes

**Figure 7:**
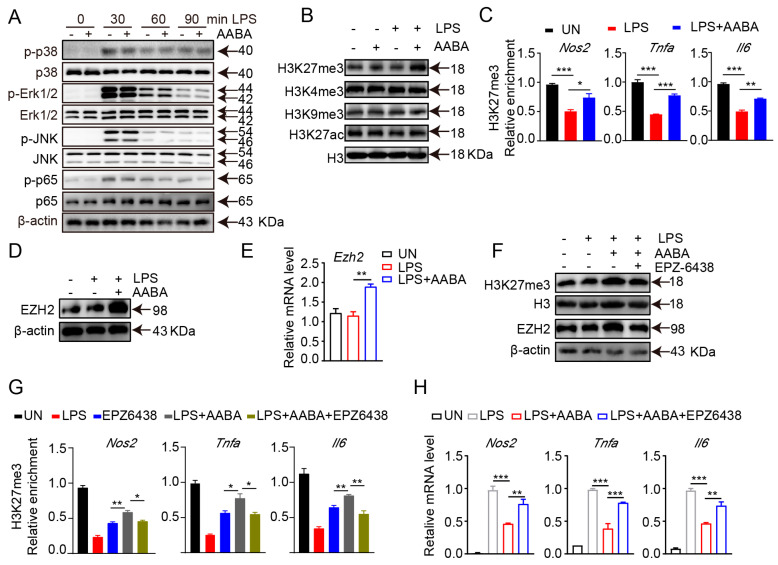
AABA enhances H3K27me3 occupancy on M1 macrophage-associated inflammation genes. LPS (100 ng/mL), AABA (1 mM), and EPZ6438 (10 μM) were used. (**A**) Western blot images of p38, Erk1/2, JNK, NF-κB, and their phosphorylation. (**B**) Western blot images of H3K27me3, H3K9me3, H3K4me3, and H3K27ac. (**C**) Enrichment of H3K27me3 at the promoter of *Nos2*, *Tnfa*, and *Il6*. (**D**) Western blot image of EZH2. (**E**) *Ezh2* mRNA level. (**F**) Western blot images of H3K27me3 and EZH2. (**G**) Enrichment of H3K27me3 at the promoter of *Nos2*, *Tnfa*, and *Il6*. (**H**) *Nos2*, *Tnfa*, and *Il6* mRNA levels. * *p* < 0.05, ** *p* < 0.01, *** *p* < 0.001.

In addition to metabolic reprogramming, MAPK and NF-κB signaling transduction are involved in the regulation of macrophage polarization [21]. However, we showed that AABA treatment did not repress LPS-induced MAPK in three members, extracellular signal-regulated kinase (ERK)1/2, c-Jun N-terminal kinase (JNK), p38 and NF-κB activation, indicating that AABA affected M1 macrophage activation through other mechanisms. (Figure 7A). 

The functional plasticity of macrophage is closely modulated by transcriptional reprogramming, which is implemented by altering the chromatin accessibility and epigenetic landscape [9]. Therefore, we speculated that AABA may inhibit the activation and function of M1 macrophages by modulating their epigenetic modifications. We examined the expression of H3K27me3, H3K27ac, H3K4me3, and H3K9me3 in AABA-treated and control BMDMs. The results showed that AABA enhanced H3K27me3, but not H3K27ac, H3K4me3, or H3K9me3 (Figure 7B). H3K27me3 is an epigenetic marker that functions as a repressor of gene expression and limits the expression of inflammatory genes in M1 macrophages [1]. Chromatin immunoprecipitation quantitative polymerase chain reaction (ChIP-qPCR) analysis revealed that AABA treatment enriched H3K27me3 at the promoter regions *Nos2, Tnfa*, and *Il6* (Figure 7C). EZH2 is a lysine methyl transferase that catalyzes H3K27me3 modification. AABA promoted the mRNA and protein expression of EZH2 (Figure 7D,E). Moreover, the EZH2 inhibitor EPZ6438 reversed the AABA-induced increases in EZH2 and H3K27me3 expression. (Figure 7F). Additionally, EPZ6438 suppressed the enrichment of H3K27me3 at the promoter regions of *Nos2, Tnfa*, and *Il6,* and maintained the expression of these genes in response to AABA treatment (Figure 7G,H). Collectively, these data suggest that AABA promotes the H3K27me3 occupancy on inflammatory genes in M1 macrophages by increasing the expression of EZH2 to inhibit activation and function of M1 macrophages.

## 3. Discussion

Macrophages occupy significant parts in the development of inflammatory diseases, as highlighted by numerous studies. In murine sepsis, the hyperactivation of macrophages can trigger a cytokine storm that exacerbates tissue damage and worsens the disease [6]. Similarly, in murine colitis, compromised intestinal barriers can lead to the recruitment and infiltration of inflammatory macrophages, perpetuating inflammation [33]. Based on these observations, we hypothesized that AABA supplementation might serve as a regulator to constrain LPS-induced macrophage polarization and function. Our subsequent experiments showed that AABA indeed had therapeutic effects in mice with sepsis induced by LPS and CLP, as well as colitis induced by DSS. Specifically, AABA inhibited the activation and function of pro-inflammatory M1 macrophages, thereby alleviating disease symptoms and promoting recovery. Our research indicates that AABA can potentially serve as a valuable therapeutic agent for a range of inflammatory diseases.

The immunomodulatory effects of AABA have yet to be investigated. Our results demonstrated that AABA suppressed the activation and pro-inflammatory function of M1 macrophages, thereby protecting mice against LPS- and CLP-induced sepsis and DSS-induced inflammatory bowel disease. In the beginning stage of sepsis, macrophages act as the primary defense mechanism by recognizing and engulfing invading pathogens [34]. They also release various mediators to promote the activation of other immune cells and chemotaxis towards the site of infection. Dendritic cells can carry molecules recognized as antigens and present them to T lymphocytes, which activate specific immune responses [35]. Natural killer cells can secrete large amounts of cytotoxic substances and various chemokines that attract other inflammatory cells to the site of infection [36]. Similarly, colitis in mice involves the cooperation of various immune cells, including neutrophils, macrophages, dendritic cells, and T cells [37]. Overall, our study supports the immunomodulatory effects of AABA in suppressing M1 macrophage activation and pro-inflammatory function, leading to protection against sepsis and inflammatory bowel disease in mice. It suggests that AABA is poised to be used as a therapeutic treatment for immune-related disorders. Further investigations are required to understand the impacts of AABA on other immune cells and to fully elucidate its mechanism of action. These findings shed light on the role of AABA in immune response modulation and hold promise for future therapeutic interventions.

Numerous research has highlighted the importance of immunometabolism in regulating the activation and function of immune cells, with metabolic pathways shown to modulate macrophage polarization and function [10,16,38]. For instance, it has been observed that LPS stimulation induces the activation of M1 macrophages, characterized by enhanced glycolysis and a broken tricarboxylic acid (TCA) cycle [10]. Subsequent metabolic shifts result in fluctuations in intracellular metabolites, which may further regulate macrophage function [39]. Here, we explored the effects of AABA on the metabolic pathways of LPS-stimulated macrophages. Specifically, we hypothesized that AABA level might decrease following LPS stimulation, given that AABA is a byproduct of four amino acids whose levels were significantly reduced in macrophages exposed to LPS (methionine, threonine, serine, and glycine). Our results showed that AABA treatment had multiple effects on LPS-stimulated macrophages. Notably, AABA inhibited glycolysis while enhancing oxidative phosphorylation, resulting in the restoration of mitochondrial function. While M1 macrophages typically rely on glycolysis to support their inflammatory response, we did not observe any change in IL-1β production following AABA treatment. AABA was previously reported to increase intracellular levels of GSH [25], which is vital for IL-1β production [19]. However, we did not find a notable alteration in IL-1β production of M1 macrophages after AABA treatment. This may be because of the slightly different mechanisms through which AABA regulates cellular functions in different cell types. Metabolomics analysis further revealed that AABA could enhance the metabolism pathway of multiple amino acids in M1 macrophages, including arginine, glutamine, and glutamate. Interestingly, both M1 and M2 macrophages utilize arginine to support their polarization via iNOS and arginase-1, respectively [40]. We observed that AABA treatment led to decreased expression of iNOS while enhancing the arginine metabolism pathway, raising the possibility that AABA may promote M2 polarization by facilitating arginase-1 activity. Additionally, glutamine has been shown to feed TCA in the form of glutamate and α-KG, promoting M2 polarization and inhibiting the M1 macrophage inflammation response [23,41]. Our results suggest that AABA may reverse M1 macrophage polarization towards an M2 phenotype, although this requires further investigation.

Mechanically, our findings suggest that AABA regulates macrophage polarization via epigenetic pathways, rather than via the MAPK and NF-κB signaling routes. AABA promotes the expression of the transcription repressor EZH2 to encourage the enrichment of H3K27me3 at promoter regions of M1 marker genes, such as *Nos2*, *Tnfa*, and *Il6*. Therefore, we examined histone modification markers such as H3K27me3, H3K27ac, H3K4me3, and H3K9me3 in AABA-treated and control BMDMs. The results showed that AABA enhanced H3K27me3 at the promoter regions of *Nos2*, *Tnfa*, and *Il6*. EZH2, the catalytic component of polycomb repressive complex 2, is considered to facilitate the trimethylation of histone H3 at lysine 27 via methyltransferase activity [42,43]. As previously reported, EZH2 can suppresses the inflammatory response of intestinal epithelial cells by inhibiting the expression of various inflammatory factors [44,45]. Here, we found that AABA-induced H3K27me3 enrichment was EZH2-dependent. AABA treatment promoted the expression of EZH2, and the EZH2 inhibitor EPZ6438 abolished the enrichment of H3K27me3 at the promoter regions of *Nos2*, *Tnfa*, and *Il6*.

Gamma- aminobutyric acid (GABA) is an isomer of AABA. They share the same chemical formula, C4H9NO2, but differ in the position of the amino group [46]. GABA is a naturally occurring amino acid that serves as the principal inhibitory neurotransmitter in the central nervous system [47]. GABA has also been reported to limit the release of pro-inflammatory cytokines in DSS-induced colitis [48]. Currently, GABA has been commercially available as a nutritional supplement that can improve sleep and memory. Additionally, its potential clinical applications in addressing high blood pressure and promoting growth hormone secretion have been extensively studied [49]. On the other hand, abnormally high levels of AABA have been found to indicate and partially predict the progression to a severely dysregulated metabolic pattern in sepsis [24]. Here, our findings demonstrated that administering AABA significantly improves the survival of septic mice and mitigates the severity of colitis in mice. Notably, oral administration of AABA has been reported to activate AMP-activated protein kinase to increase glutathione levels in circulating and myocardial functions, thus protecting mice from doxorubicin-induced cardiomyopathy in mice [25]. These findings the indicate potential value of AABA for disease alleviation. However, further studies are needed to validate its safety and clinical application.

## 4. Materials and Methods

### 4.1. Mice and Generation of BMDMs

C57BL/6J mice were purchased from Beijing Vital River Laboratory Animal Technology for in vitro and in vivo experiments. Bone marrow-derived macrophages (BMDMs) were isolated from 7-week-old C57BL/6J female mice and treated with erythrocyte lysis solution before neutralization with DMEM medium. Cells were cultured with 50 ng/mL of macrophage colony stimulating factor (M-CSF) and 10 mL of DMEM containing 10% FBS in 100 mm cell culture disks. Cell culture medium and M-CSF were refreshed every two days. On day 7, the BMDMs were digested with accutase solution and plated in 12-well plates at a density of 1 × 10^6^/well. After 12 h of cell plating, cells were treated with LPS (100 ng/mL) or LPS (100 ng/mL) plus AABA (100 or 1000 μM) for 24 h, then the medium and cells were collected for the subsequent experiments. 

### 4.2. LPS-Induced Sepsis Model

AABA and LPS were fully dissolved in phosphate-buffered saline (PBS) and stored at −80 °C before use. The 8-week-old female C57BL/6J mice were divided into the following groups: PBS (+) LPS (−), AABA (+) LPS (−), PBS (+) LPS (+), AABA (+) LPS (+). Groups AABA (+) LPS (+) and PBS (+) LPS (+) were injected intraperitoneally with 20 mg/kg LPS, followed by an intraperitoneal injection of 75 mg/kg AABA or an equivalent volume of PBS. Groups AABA (+) LPS (−) and PBS (+) LPS (−) were injected intraperitoneally with 75 mg/kg AABA or an equivalent volume of PBS as a control. Disease progression was determined by two blinded investigators, and mice were monitored every 1 h to record the time of death. According to experimental requirements, tubes containing anticoagulant were prepared to collect mice’s ocular blood. The blood samples were centrifuged at 10,000 rpm for 20 min, then the supernatants were transferred to new tubes. The sera samples were stored at −80 °C before the subsequent experiments. Next, the peritoneal macrophages were collected using Trizol or radio-immunoprecipitation assay (RIPA) buffer for the next analysis of gene or protein. 

### 4.3. Cecal Ligation and Puncture (CLP)

The CLP procedure steps refer to a previous study [50]. The mice were divided into three groups: sham, CLP + PBS, and CLP + AABA. The AABA was fully dissolved in PBS and stored at −80 before use. After the CLP procedure, the mice were treated with 75 mg/kg AABA or an equivalent volume of PBS as a control. After 12 h of CLP procedure, the mice sera of three groups were collected and stored at −80 °C before the subsequent detection of cytokines and mediators. The peritoneal macrophages were collected using Trizol or RIPA reagent for the next analysis of gene or protein. For survival rate, the disease progression was determined by two blinded investigators, and mice were monitored every day to record the time of death. 

### 4.4. The Development and Evaluation of DSS-Induced Colitis in Mice

DSS was dissolved in filtered water and prepared to a concentration of 2.5% (*w*/*v*). The C57BL/6J mice were divided into the following groups: PBS (+) DSS (−), AABA (+) DSS (−), PBS (+) DSS (+), AABA (+) DSS (+). Group AABA (+) DSS (+) and PBS (+) DSS (+) were given water containing 2.5% DSS for the first 5 days, then changed to normal water for the last 3 days. Throughout the 8 days, mice in the AABA (+) DSS (+/−) group were given daily intraperitoneal injections of 75 mg/kg of AABA. The colitis progression was assessed by recording the body weight of each mouse and calculating the body weight percentage change daily. The Disease Activity Index (DAI) was measured with reference to previous study [51].

### 4.5. Peritoneal Macrophage Isolation

The mice were sacrificed after the termination of the experiment. Next, the mice were intraperitoneally injected with 5 mL of ice-cold PBS. After massaging the abdomen of the mice for 3–5 min, the intraperitoneal fluid was collected in a 15 mL tube. The cell suspension was centrifuged at 2000 rpm at 4 °C for 3 min. The supernatant was discarded, and 5 mL of erythrocyte lysate was added and mixed well at room temperature for 5 min. The reaction was terminated with PBS. Next, the cells were resuspended with 5 mL of DMEM medium containing 10% FBS and transferred to a 60 mm culture dish. The cells were cultured for 2 h in a 37 °C incubator. Finally, the unadhered cells were discarded, and peritoneal macrophages were collected with a scraper. The peritoneal macrophages were collected using Trizol or RIPA reagent for the next analysis of gene and protein. In addition, peritoneal macrophages were collected using PBS for next flow cytometry using FACS Canto II (BD Biosciences, San Jose, CA, USA).

### 4.6. Intestinal Macrophage Sorting

Colorectal intestinal tissue was removed and cut into pieces 4–5 cm in length. The intestinal tissue was washed with ice-cold PBS in 4 mL tube. Each tube had 3 mL of intestinal epithelial digestion solution (EDTA 0.4 mg/mL, DTT 0.2 mg/mL) added and was placed on a 37 °C shaker at 200 rpm for 40 min. The intestinal tissue was washed 3 times with ice-cold hanks solution. Next, the intestine was cut to a 3–4 mm length. Each tube had 3 mL intestinal lamina propria digestion solution (Type IV collagenase 0.4 mg/mL, DNase 0.2 mg/mL, FBS 0.5%, *v*/*v*) added and was then placed on a 37 °C shaker at 200 rpm for 60 min. The intestine was grinded and filtered through a 100 μm cell strainer into 50 mL centrifuge tube. The suspension was centrifuged at 2000 rpm for 5 min. The supernatant was discarded and the cells were resuspended with 4 mL of 40% percoll in a 15 mL tube. The cells were centrifuged with 2200 rpm (acceleration 3, deceleration 3). Next, the single-cell suspension was incubated with anti-CD16/32 antibody on ice for 10 min. At last, cells were stained with the following antibodies: Fixable Viability, CD45, F4-80, CD11b. Finally, the F4-80^+^ CD11b^+^ intestinal macrophages were collected using FACSAria II (BD Biosciences, Franklin Lakes, NJ, USA).

### 4.7. Measurement of IL-6 and TNF-α

A total of 100 μL of cell culture supernatant or mouse sera was added to the detection plate, followed by the addition of 50 μL of detection antibody. The solution was thoroughly mixed and then incubated at 37 °C for 90 min. Afterwards, the supernatant was discarded, and each well was washed four times with 300 μL of washing buffer for one minute per wash. Subsequently, 100 μL of Streptavidin-HRP was added to each well and incubated at 37 °C for 30 min. The liquid was then discarded, and each well was washed four more times with 300 μL of washing buffer for one minute per wash. Next, 100 μL of TMB buffer was added to each well and incubated at 37 °C for 10–20 min. Finally, 100 μL of stop solution was added to each well to terminate the reaction. Within 10 min after the reaction was stopped, the OD value was read using a measurement wavelength of 450 nm (Thermo, Multiskan Sky High, Waltham, MA, USA).

### 4.8. Measurement of NO

Griess Reagent was applied for NO detection. Before the experiment began, Griess Reagent I and II were brought to room temperature. A total of 50 μL of cell culture supernatant or mouse sera was added to a 96-well plate. Then, 50 μL of Griess Reagent I was added to each well, followed by the addition of another 50 μL of Griess Reagent II. The OD value was read using a wavelength of 540 nm (Thermo, Multiskan Sky High, Waltham, MA, USA).

### 4.9. Measurement of ALT and AST

The 5 μL of mouse sera was incubated with 20 μL of substrate solution for aspartate aminotransferase or alanine aminotransferase at 37 °C for 30 min. Subsequently, 20 μL of 2,4-dinitrophenylhydrazine solution was added to the well and incubated at 37 °C for 20 min. After that, 200 μL of 0.4 mol/L NaOH was added, followed by gently shaking at room temperature for 15 min. Finally, the OD value was measured using a wavelength of 510 nm (Thermo, Multiskan Sky High, Waltham, MA, USA).

### 4.10. ECAR and OCR Analysis

Macrophages were collected and seeded at 40,000 cells/well in Agilent 24-well plates. Next, cells were treated with LPS or LPS plus 100 μM AABA for 24 h. Each well contained 500 µL of XFp-DMEM medium, 1 mM pyruvate, 2 mM glutamine, and 25 mM glucose (ECAR medium 0 mM). The plate was incubated in a CO_2_-free incubator at 37 °C for 30 min before starting the assay using a Seahorse XFp analyzer (Agilent Technologies, Santa Clara, CA, USA). For oxygen consumption rate (OCR): oligomycin (oligo, 10 µM), FCCP (10 µM), and rotenone/antimycin A (R + A, 5 µM) were added sequentially. For extracellular acidification rate (ECAR): glucose (Glu, 10 mM), oligomycin (5 μM), and 2-DG (50 mM) were added sequentially. The rates of OCR and ECAR were calculated using the Wave software V2.4.0 (Agilent Technologies, Santa Clara, CA, USA) and normalized to total cell number.

### 4.11. LC-MS/MS

Sample preparation: untreated BMDMs and LPS-stimulated BMDMs (36 h) were collected in tubes and then centrifuged at 1500 rpm for 5 min. The supernatant was discarded and cells were quickly frozen with liquid nitrogen. LPS-stimulated BMDMs and LPS plus 1mM AABA-treated BMDMs (24 h) were collected. The supernatant was discarded and cells were quickly frozen with liquid nitrogen. Next, the preparation procedure for cell samples was handed as previously described [52]. Subsequently, the LC-MS/MS was performed using Agilent 1290 Infinity II series (Agilent Technologies, Santa Clara, CA, USA), and the steps were consistent with previous study [53].

### 4.12. Reagents and Complete Methods

All reagents used in the text are shown in the Supplementary Materials. The complete experimental methods are provided in the Supplementary Materials**.**

### 4.13. Data Analysis 

Statistical analysis was performed using Prism 8.0 (GraphPad, San Diego, CA, USA) software. Flow Jo V10 software (BD Biosciences, San Jose, CA, USA) was used to analyze the flow cytometry data. Statistical significance was determined using Student’s *t*-tests or two-sided log-rank (Mantel–Cox) test. * *p* < 0.05, ** *p* < 0.01, *** *p* < 0.001, ns: no significance. Data are presented as mean ± s.e.m.

## 5. Conclusions

**Figure 8:**
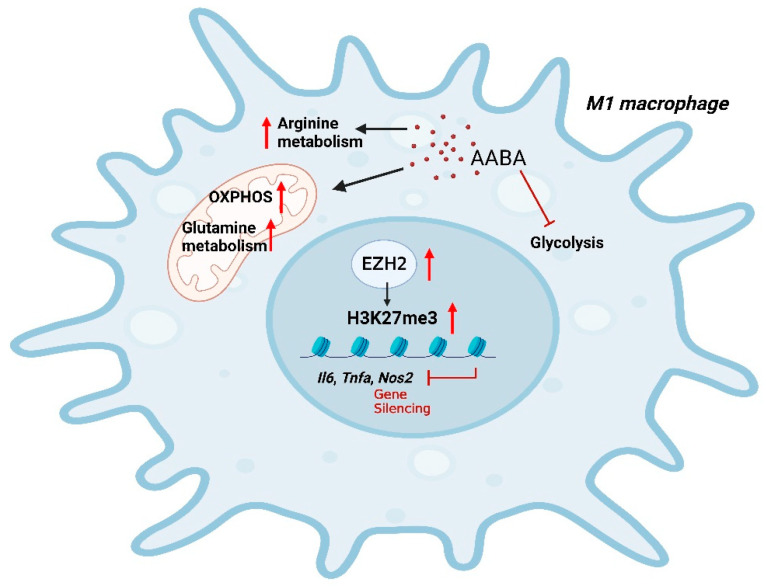
A schematic diagram of AABA constraining M1 macrophage polarization. AABA enhanced OXPHOS and arginine, glutamine metabolism while inhibiting glycolysis in M1 macrophages. Furthermore, AABA promoted the expression of the methylation transferase EZH2, leading to an enrichment in H2K27me3 modification at the promoter regions of M1 macrophage-associated genes. The diagram was created using Biorender.

In brief, our findings indicate that AABA can effectively suppress inflammatory gene expression and cytokine secretion, thereby limiting the polarization and function of M1 macrophages. Moreover, AABA treatment can improve the survival rate of mouse sepsis and alleviate the progression of mice colitis by ameliorating the inflammatory response of M1 macrophages. Notably, AABA promoted OXPHOS and arginine and glutamine metabolism, while inhibiting glycolysis in M1 macrophages. Additionally, we observed that AABA upregulated EZH2 expression, further enhancing trimethylation of histone H3 K27 at the promoter regions of M1 macrophage inflammatory genes to constrain M1 polarization (Figure 8). In summary, our research examines the immunomodulatory effects of AABA on M1 macrophages and suggests its potential for treating inflammatory diseases mediated by M1 macrophages.

## Data Availability

Data will be made available on request.

## Supplementary Materials

The supporting information can be downloaded at: https://www.mdpi.com/article/10.3390/ijms241310444/s1
