# Supplementary material for: α-Aminobutyric Acid Constrains Macrophage-Associated Inflammatory Diseases through Metabolic Reprogramming and Epigenetic Modification
Source: Int J Mol Sci. 2023 Jun 21;24(13):10444. doi: 10.3390/ijms241310444 (PMC10342172; doi:10.3390/ijms241310444)
Supplement: Supplementary file 1 [file ijms-24-10444-s001.zip › ijms-2450692-supplementary.pdf]

Supplementary Information for

α-Aminobutyric Acid Constrains Macrophage-Associated Inflammatory Diseases through Metabolic Reprogramming and Epigenetic Modification

Fei Li [1], Yuting Xia [2], Shijie Yuan [1], Xiaorong, Xie [1], Lin Li [3], Yuan Luo [1], Qiuyang Du [1], Yuqi Yuan [1] and Ran He [1,*]

[1] Department of Immunology, School of Basic Medicine, Tongji Medical College, Huazhong University of Science and Technology, Wuhan 430032, China; feili@hust.edu.cn (Fei Li)

[2] Department of Dermatology, Tongji Medical College, Huazhong University of Science and Technology, Wuhan 430032, China

[3] State Key Laboratory of Oncology in South China, Sun Yat-sen University Cancer Center, Guangzhou 510060, China

* Correspondence: ranhe@hust.edu.cn (Ran He).

This PDF file includes:

1. Reagents

2.Supplementary methods

1. Reagents

Reagents	Identify	Source	City and Country
α-aminobutyric acid (AABA)	A1879	Sigma	Garlsbad, CA, USA
anti-β-actin rabbit	D4E3M	CST	Danvers, MA, USA
anti-iNOS rabbit	D6B6S	CST	Danvers, MA, USA
anti-p38 rabbit	D13E1	CST	Danvers, MA, USA
anti-Phospho-p38 rabbit	D3F9	CST	Danvers, MA, USA
anti-JNK rabbit	9252	CST	Danvers, MA, USA

anti-Phospho-JNK rabbit	81E11	CST	Danvers, MA, USA
anti-p44/42 rabbit	137F5	CST	Danvers, MA, USA
anti-Phospho-p44/42 rabbit	D13.14.4E	CST	Danvers, MA, USA
anti-p65 rabbit	D14E12	CST	Danvers, MA, USA
anti-Phospho-p65 rabbit	93H1	CST	Danvers, MA, USA
anti-H3K27me3 rabbit	C36B11	CST	Danvers, MA, USA
anti-H3K4me3 rabbit	C42D8	CST	Danvers, MA, USA
anti-H3K9me3 rabbit	D4W1U	CST	Danvers, MA, USA
anti-H3K27ac rabbit	D5E4	CST	Danvers, MA, USA
mouse anti-CD80	16-10A1	BioLegend	San Diego, CA, USA
mouse anti-CD86	GL-1	BioLegend	San Diego, CA, USA
mouse anti-F4-80	BM8	BioLegend	San Diego, CA, USA
Fixable Viability Dye	65-0866-14	Thermo	Garlsbad, CA, USA
anti-CD16/32	93	BioLegend	San Diego, CA, USA
mouse anti-CD45	S18009D	BioLegend	San Diego, CA, USA
mouse anti-CD11b	M1/70	BioLegend	San Diego, CA, USA
TMRE	T669	Thermo	Garlsbad, CA, USA
mito tracker Deep Red	M22426	Invitrogen	Carlsbad, CA, USA
RIPA Lysis Buffer	P0013K	Beyotime	Wuhan, China
Phenylmethanesulfonyl fluoride (PMSF)	G2002	Servicebio	Wuhan, China
phosphatase inhibitor cocktails	K1015	APExBIO	Boston, MA, USA
Protein Free Rapid Blocking Buffer	PS108	Epizyme	Shanghai, China

HRP-linked anti-rabbit	GB23303	Servicebio	Wuhan, China
6× protein loading buffer	DL101-02	TransGen	Beijing, China
PageRuler Prestained Protein Ladder	26616	Thermo	Garlsbad, CA, USA
BeyoECL Moon	P0018FS	Beyotime	Wuhan, China
Goldenstar™ RT6 cDNA Synthesis Mix	TSK314S	TSINGKE	Beijing, China
DSS	60316ES60	YEASEN	Shanghai, China
Dithiothreitol (DTT)	BS110	biosharp	Anhui, China
Ethylene Diamine Tetraacetic Acid (EDTA)	BS107	biosharp	Anhui, China
Type IV collagenase	BS165	biosharp	Anhui, China
DNase Ⅰ	BS137	biosharp	Anhui, China
Percoll	17089101	Cytiva	Marlborough, MA, USA
LPS	L2880	Sigma	Garlsbad, CA, USA
Accutase	00-4555-56	Thermo	Garlsbad, CA, USA
DMEM	8121451	Gibco	Garlsbad, CA, USA
M-CSF (macrophage colony stimulating factor)	315-02	Peprotech	Cranbury, NJ, USA
FBS	SA101.02	Cellmax	Beijing, China
LPS (*in vitro*)	tlrl-eblps	InvivoGen	San Diego, CA, USA
EPZ6438	S7128	Selleck	Houston, TX, USA
TRIzol reagent	R0016	Beyotime	Wuhan, China
IL-6 ELISA kit	1210602	DAKEWE	Beijing, China
TNF-α ELISA kit	1217202	DAKEWE	Beijing, China
ALT	C010-2-1	NJJCBIO	Nanjing, China

AST	C009-2-1	NJJCBIO	Nanjing, China
NO	S0021S	Beyotime	Wuhan, China
CCK8 kit	C0037	Beyotime	Wuhan, China
XF Glycolysis Stress Test Kit	103020-100	Agilent	Santa Clara, CA, USA
XF Cell Mito Stress Test Kit	103015-100	Agilent	Santa Clara, CA, USA

2.Supplementary methods

RNA purification, RT-PCR and real-time PCR

TRIzol reagent was used to extract cellular and tissue RNA. Next, Goldenstar™ RT6 cDNA Synthesis Mix was mixed with 1 μg of RNA for 15 min at 50 ℃ and 5 min at 85 ℃. At the end of the reaction, the cDNA, TSINGKE Master qPCR Mix and 10 pmol of forward and reverse primers were mixed and subsequently analyzed for target gene mRNA levels. After cDNA amplification with Biorad CFX connect thermocycler (Biorad, CA, USA)., the relative quantification of mRNA was normalized to β-actin (*Actb*). The primers are listed as follows: *Actb* (forward: 5′-TCCATCATGAAGTGTG-ACGT-3′), reverse: 5′-TACTCCTGC-TTGCTGATCCAC-3′); *Nos2* (forward: 5′-GAGACAGGGAAGTCTGAAG-CAC-3′, reverse: 5′-CCAGCAGTA-GTTGCT-CCTCTTC-3′); *Tnfa* (forward: 5′-CAGGCGGTGCCT-ATGTCTC-3′, reverse: 5′-CGATCACCCCGAAGTTC-AGTAG-3′); *Il6* (forward: 5′-CTGCAAGAGACTT-CCATCCAG-3′), reverse: 5′-AGTGGTATAGACAGGT-CTGTTGG-3′).

Chromatin immunoprecipitation (ChIP)

At 24 h of cell treatment, 270 μl of 37% paraformaldehyde was add to 10 ml cell culture medium, mixed well, and incubated for 10 min at 37 ℃ for fixation and cross-linking. Next, the reaction was terminated by adding glycine and incubating at room temperature for 5 min. Afterwards, 1×10^7 cells were collected in SDS lysis buffer and was fragmented by sonication. Chromatin was then pulldown with 1 μg of anti-H3K27me3 or IgG control antibody at 4 °C overnight. Next, the proteins and chromatin were uncross-linked at 65 °C for 4 hours. The DNA was then eluted and purified, and finally analyzed using a chromatin immunoprecipitation (ChIP)-qPCR procedure with Biorad CFX connect thermocycler (Biorad, CA, USA). DNA were first normalized to control IgG, followed by normalized to Input DNA. ChIP-qPCR primers are used as follows: Nos2 (forward: 5′-AGTGTTCCTGGCTTATCCT-3′, reverse: 5′- ATGTCCTACCTGGGTGTG-3′); Tnfa (forward: 5′-GTTG-TAGAG-TGTTCAATGAGAG-3′, reverse: 5′-GCGTCTTGTCTGATCTGTT -3′); Il6 (forward: 5′-CACAGGCGATTAGAGTAGAT-3′, reverse: 5′-GATTGT-GATCCTGAGAGTGT-3′).

Immunoblotting

Cells were lysed using RIPA lysis solution containing 1× PMSF and phosphatase inhibitor cocktails, mixed with 6× protein loading buffer, heated at 100 ℃ for protein

denaturation, followed by a short centrifugation. Next, proteins were separated by 10% gels SDS-PAGE, then blotted onto 0.45 μm PVDF membranes. Next, the Protein Free Rapid Blocking Buffer was used to block the membrane for 30 min at room temperature. The membrane was incubated with the antibodies in Table S5 at 4 °C overnight. Membranes were then washed 3 times for 5 min, and incubated with HRP-linked anti-rabbit secondary antibodies, followed by analysis of exposure with ChemiDoc XRS (Biorad, CA, USA)

Flow cytometry

1 ml of FBS was dissolved in 100 ml of PBS for FACS buffer. Cells were seeded in 96-well plates at a density of 1×10^6 cells, followed by incubation with anti-CD16/32 antibody on ice for 10 min. Then, the cells were washed with ice-cold FACS buffer, followed by incubation with cell surface antibodies (Fixable Viability Dye, f4-80, CD11b, CD80, CD86) for 30 min at 4 °C. The cells were then washed with ice-cold FACS buffer three times. Finally, the cells were resuspended with FACS buffer and detected by BD FACS CANTO or BD FACS LSR II (BD Biosciences, CA, USA). and data were analyzed using FlowJo V10 software (BD Biosciences, CA, USA).

Mitochondial membrane potential assay

Cells were collected and then washed twice with ice-cold PBS. Cells were incubated with 50 nM TMRE, 50 nM Mito Deep Red for 30 min at 37 °C. At last, cells were washed by ice-cold PBS twice, then cells were resuspended with PBS and detected by flowmetry within 1 h.

Bactericidal ability test

The untreated BMDMs, LPS-treated BMDMs and LPS + 100 or 1000 μM AABA-treated BMDMs for 24 h were co-cultured with E. coli at a ratio of 1:10 for 6 h and then collected and coated in ampicillin-containing agarose solid medium for 24 h, then colony- forming units were counted.
